# Challenges in the assessment of total fluid intake in children and adolescents: a discussion paper

**DOI:** 10.1007/s00394-018-1745-7

**Published:** 2018-06-12

**Authors:** Janet Warren, Isabelle Guelinckx, Barbara Livingstone, Nancy Potischman, Michael Nelson, Emma Foster, Bridget Holmes

**Affiliations:** 1 FirstStop Nutrition Ltd, Oxford, UK; 2 Danone Nutricia Research, Route Départementale 128, 91767 Palaiseau, France; 3 Nutrition Innovation Centre for Food and Health, Ulster University, Coleraine, UK; 4 Office of Dietary Supplements, National Institutes of Health, Bethesda, USA; 5 Public Health Nutrition Research Ltd, King’s College London, London, UK; 6 Human Nutrition Research Centre, Newcastle University, Newcastle, UK

**Keywords:** Dietary assessment, Beverages, Fluid intake, Water, Hydration, Children

## Abstract

**Purpose:**

In recent years, evidence has emerged about the importance of healthy fluid intake in children for physical and mental performance and health, and in the prevention of obesity. Accurate data on water intake are needed to inform researchers and policymakers and for setting dietary reference values. However, to date, there are few published data on fluid or water intakes in children. This is due partly to the fact that drinking water is not always reported in dietary surveys. The aim of this paper is to review the current status of the literature and highlight the challenges of assessing total fluid intake in children and adolescents.

**Results:**

From the dietary assessment literature it is apparent that children present unique challenges to assessing intake due to ongoing cognitive capacity development, limited literacy skills, difficulties in estimating portion sizes and multiple caregivers during any 1 day making it difficult to track intakes. As such, many issues should be considered when assessing total fluid intakes in children or adolescents. Various methods to assess fluid intakes exist, each with its own strengths and weaknesses; the ultimate choice of method depends on the research question and resources available. Based on the literature review, it is apparent that if the research focus is to assess only fluid intake, a fluid-specific method, such as a diary or record, appears to be a feasible approach to provide an accurate estimate of intakes.

## Introduction

The association between hydration, water and beverage intake has become an important area of research as evidence of the link between water intake and physical disease and cognitive performance increases [1, 2]. In 2011, the European Food Safety Authority stated that water is the “basic requirement of all living things” as it ensures maintenance of normal physical and cognitive functions [3]. Since then, recent studies have confirmed the impact of fluid intake on physical and cognitive performance in children, and regular drinking (including during lessons) has been recommended, based on empirical evidence obtained from two studies [4–6].

Accurate data on water intake are needed to inform policy and for setting dietary reference values. Reducing or limiting sugar-sweetened beverages intake has been targeted as a public health prevention measure for more than a decade [7]. The Institute of Medicine in the US concluded in 2004 that it was not possible to set an estimated average requirement for water due to the wide variation in requirements and insufficient evidence. They, therefore, set “adequate intakes” based on median intakes of total water intake (water from food and fluid) observed in national surveys [8]. The European recommendations for water intake were partly informed by observed intakes in the population, along with desirable urinary osmolality values and desirable water volumes per unit of energy consumed [9]. Thus, the measurement of water intake from specific sources as well as total fluid intake (TFI) is important from a public health perspective.

Historically, water intake was overlooked in most dietary surveys or at least not given emphasis since water does not contribute to energy intake [10]. This is a similar observation for beverage intake. In 2014, Özen and colleagues systematically reviewed and published beverage intakes from peer-reviewed publications [11]. Three-quarters of articles were excluded on the basis of insufficient data on beverage intake. Additionally, 63% of the reviewed surveys among children did not report drinking water [11]. This issue has been addressed in the US; both the NHANES (National Health and Nutrition Examination Survey) and ASA24 (Automated Self-Administered 24-h Recall) include a question about water intake as a ‘frequently forgotten’ item.

The dietary sources of water are food moisture and fluids (e.g. drinking water and the water content of soft drinks, juices, milk, coffee, tea and alcohol). Knowledge of the contribution of food moisture to total water intake has increased in recent years. It varies between countries depending on local consumption habits. The contribution is estimated to be 20–30% in Europe, and 40% in China where the consumption of soups and liquid-based meals is higher [1]. The contribution of food to total water intake is estimated to be 51% in Japan [12], 27% and 36% in the UK and France, respectively [13], 20% in USA [8] and 31% in Ireland [14]. Given that some foods can make a significant contribution to total water intake, obtaining an account of all food and fluids consumed will give the most complete picture of total water intake providing that there is an acceptable level of accuracy in the dietary reporting. Despite food moisture data being available in many food composition tables and databases, it is rarely calculated for population surveys [15]. Therefore, an assumption is usually made about the percentage of food moisture in the diet which may introduce a bias or inaccuracies.

This paper aims to highlight the key issues and challenges that need to be considered when assessing total fluid intake in children and adolescents. It is intended to be a starting point resource for researchers interested in assessing total fluid intake in children and adolescents. The following terminology is employed: total water refers to water from all sources including food; fluid intake refers to the consumption of drinking water and all other beverages [9].

## Methodology

A literature review was undertaken in Web of Science and PubMed searching for papers on dietary assessment methods/methodology, validation, food records, recalls or food frequencies, fluid/water intake, contribution of food to water content of the diet, portion size estimation and use of technology in children and/or adolescents. Literature from January 2011 to August 2017 was searched; this built on a previous literature review carried out by some of the authors and presented at the International Conference on Diet and Activity Methods in 2011 [16].

This literature review was a comprehensive but not systematic search. Papers identified have not been rated or scored according to level of quality, validation, or any other factors. Given that the measurement and validation of fluid intake assessment is relatively new, some recent examples of studies in adults are given to inform the reader and give an indication of the direction of research. With respect to portion size estimation and use of technology the general dietary assessment literature is referred to as a guide to considerations for the assessment of fluid intake.

## Challenges and considerations of assessing total fluid intake in children and adolescents, with reference to the dietary assessment literature

Currently, the assessment of fluid intake is overwhelmingly based on traditional dietary assessment methodologies, because of lack of evidence to support proof of primacy of one method over another [17].

The choice of method depends on the research question and objective of the study along with characteristics of the study participants. For readers seeking more detailed information or a catalogue to make a guided choice of validated dietary assessment tools, please refer to the DAPA (Diet and Physical Activity) Measurement Toolkit (UK) [18], the National Cancer Institute’s Dietary Assessment Primer (USA) [19], the DIET@NET which is an abbreviation of DIETary Assessment Tools NETwork and was funded by the Medical Research Council (UK) [20], and the Australasian Child and Adolescent Obesity Research Network (ACAORN) method selector (Australia) [21].

Indirect validation of a dietary assessment method may assess the level of agreement between two methods of assessment, for the parameter of interest, for example, energy intake and/or nutrient intake; objective validation can be obtained by use of biomarkers, for example, the ingestion of doubly labelled water which validates energy intake or disappearance of deuterium oxide which makes it a suitable check for total water intake.

The challenges of dietary assessment are compounded in children due to ongoing cognitive development, limited literacy skills and difficulties in estimating portion size [22]; these considerations are detailed in Table 1 by age range.

**Table 1:** Considerations for measuring total fluid intake, by age range [18, 23, 24]

Consideration	Toddlers	Pre-school	Young children	Older children	Adolescents
	1–2 years	2–4 years	5–8 years	9–12 years	13–18 years
Breastfeeding^a^/formula feeding to be assessed	Yes/no	No	No	No	No
High frequency of consumption	Yes	Yes	Yes	No	No
Need to consider regurgitation/drooling	Yes	No	No	No	No
Large amounts of wastage	Yes	Yes	Yes	Yes/no	Yes/no
Structured consumption habits	Yes	Yes	Yes/no	Yes/no	No
Ability to complete questionnaires on their own	No	No	No	Yes/no	Yes
Ability to recall information	No	No	No	Yes/no	Yes
Concept of time	No	No	No	Yes/no	Yes
Knowledge of food/drink, preparation	No	No	No	Yes	Yes
Ability to assess portion size	No	No	No	Yes/no	Yes
Multiple caregivers or locations	Yes	Yes	Yes	Yes	Yes
High amount of in-home consumption	Yes	Yes	Yes/no	Yes/no	Yes/no
Responsible for own consumption choice	No	No	Yes/no	Yes/no	Yes

^a^Exact age at which breastfeeding ceases depends on feeding habits of the child

### Toddlers (1–2 years)

Assessing intake in toddlers presents unique methodological challenges. The exact age at which the requirement to cease assessment of breastfeeding and/or formula feeding depends on the child. It is often difficult to quantify the amount a child consumes versus the amount offered. Important considerations include age of weaning, wastage at mealtimes as the toddler practices self-feeding, frequent consumption, identifying relevant portion size estimates and multiple carers [25]. Fluid intake may be easier to measure due to the use of graduated cups.

### Pre-school-age children (2–4 years)

Although there is likely to be less wastage at this age, intakes can vary considerably; over a 1-week period, however, the intake is likely to remain relatively stable. Many factors make assessing diet in this age group difficult, including consuming small amounts at frequent intervals, inability to complete questionnaires on their own, limited ability to recall information, limited food knowledge, and carers who may look after several individuals at the same time [25].

### School-age children (5–12 years)

School-age children start to make a transition from consuming food and drinks under adult supervision to taking responsibility for their own food and drink choices [25]. Parents and caregivers should be the main reporters of children’s intake until their cognitive and literacy skills are sufficiently developed [26]. Until children are 8 years (or even older), they do not have the required knowledge of all of the foods and drinks they consume to provide an accurate report of their own intake [22]. Thus, parents and carers have an important role in reporting children’s food and beverage intake. On a typical day, children may be under the care of multiple people in addition to attending school which means parental reporting may be a source of bias for foods or drinks consumed out of the home [26]. Concepts of time, memory, and attention span, knowledge of the names of foods and drinks are all needed for self-reporting, and these abilities develop usually from 8 years onward. Therefore, children aged 8–12 years are likely to require assistance from parents and carers, and/or of an interviewer-assisted administration. One review suggested that multiple-pass recalls for at least 3 days with parental proxy until 11 years of age was the most accurate method of assessing energy intake in children [27]. After 10 years of age, children can often reliably report their intakes in the past 24 h but exact skills depend on individuals and differ cross-culturally. In NHANES children begin independent reporting at 12 years of age. The ability of children below 10 years to give reliable answers by FFQ covering periods greater than 1 day is questionable [25]. Overall, the factors important to consider include changing intake habits, structured patterns, the importance of parental influence, time spent at school, child care, and friends [25]. Parents may not be reliable reporters of the child’s food and drink intake out of home, but if other carers, e.g. child minders are involved in the reporting process, levels of interest and motivation will vary [22].

### Adolescents (13–18 years)

It is well recognised in the literature that it is very difficult to assess dietary intake at this age due to rapidly changing habits and unstructured consumption patterns, less supervision by adults, less in-home eating, peer influence, variable knowledge of food/drink and preparation, responsibility for self-reporting, limited attention span, emotional and financial autonomy, and increased levels of misreporting with age [22, 23, 25].

Table 2 summarises the appropriate use of the various methods of dietary assessment by age group. In 2014, the European Food Safety Authority (EFSA) published guidance on obtaining high-quality harmonised population data for Europe [28]. For infants and children up to 10 years old, it recommended two 24-h food diaries or records followed by computer-assisted personal or telephone interview (CAPI/CATI) to clarify the data obtained.

**Table 2:** Level of appropriateness of dietary assessment methods to assess total fluid intake, by age group [18, 23, 24]

Group	Age (years)	Prospective				Retrospective				
		Test weighing	Weighed diet diary	Estimated diet diary	Checklist/diary	Single 24-h recall	Repeated 24-h recall	FFQ long	FFQ short	Diet history
Toddlers^a^	1–2	+	+	+++	+	+	++	+	+	+
Pre-school	2–4	–	+	+++	+	+	++	+	+	+
Young children	5–8	–	+	+++	+	+	++	+	+	+
Older children	9–12	–	+	+++	++	+	++	+	+	+
Adolescents	13–18	–	+	+++^b^	++	+	+++^b^	+	+	+

+++ very suitable, ++ moderately suitable, + limited suitability, – not suitable

^a^Exact age at which breastfeeding ceases depends on feeding habits of the child

^b^Methods weighted equally

A systematic review of dietary assessment methods in children found that a multiple-pass 24-h dietary recall reported by the parent was the most accurate method for reporting energy intake in 4–11 years [27]. The multiple-pass recall is a multi-staged format that is thought to be better suited to human cognition than the chronological approach [18]. For younger children aged 0.5–4 years, the authors concluded that a weighed dietary record was the best estimate of energy intake [27]. In some settings, neither of these methods is suitable for a large population-based survey given the resource implication for the repeated, possibly interviewer-administered 24-h recalls and the burden imposed by a weighed dietary record. A recent study in German toddlers has shown similar parental reports of nutrient intakes at a group level when a 3-day estimated food record was compared to a 3-day weighed record [29]. These limitations are, however, being addressed with new technology such as mobile or web-based food records (e.g. ASA24). Increasingly, dietary assessments are likely to be made on mobile and online tools as technology has the potential to overcome some of the limitations of traditional methodologies and this is discussed below.

A recent meta-analysis of 23 studies examined the correlation between self-reports and independent reports of dietary intake in dietary validation studies with children as the primary reporters [30]. The results provided evidence of a significant correlation between self-reports and independently validated reports of dietary intake [30]. Of the three methods studied, there was a tendency to under-report in 24-h recalls, the food diary was prone to both under- and over-reporting, while the FFQ tended to over-report intake. Interestingly, parental assistance significantly decreased the correlation observed, due to parental biases. Surprisingly, age was not found to be a moderator, but it was noted that only a small number of studies in the meta-analysis had been undertaken in children less than 8 years of age [30].

## State of the art in the assessment of total fluid intakes

Methods for specifically assessing total fluid intakes are in the early stages of development. A few methods have been objectively validated and are discussed at the end of the current section, although some focus on adults rather than children. Total water intake assessment presents some different issues to total dietary assessment, especially given that fluid may be consumed throughout the day rather than on a discrete occasion. Critical assessment of measurement error is challenging for fluid intake. In dietary assessment, methodologies are typically validated against reference measurements, e.g. for a particular nutrient or energy expenditure. In fluid assessment, reference methods are yet to be established. Objective comparisons of intakes with biomarkers or other physiological measures are in their infancy. Furthermore, the gold-standard biomarker for total fluid intake has yet to be determined. It is likely to include the criterion measures of, e.g. deuterium dilution and urine osmolality (a measure of hydration) [1].

A number of studies have compared their results of fluid or total water intake with objective measures. A study in Greek children aged 9–13 years assessed fluid intake over 2 days using a fluid diary and urinary hydration markers, and determined the relative risk of hypo-hydration, which was defined as a urine osmolality ≥ 800 mmol/kg water [31]. Children who failed to meet the recommendations for water intake demonstrated a risk of hypo-hydration that was 1.99–2.12 higher than those who met recommendations [31]. This demonstrated construct validity in the recordings via the diary. Both parents and children were instructed on how to complete this diary, which possibly encouraged recording completeness [31].

It is also helpful to consider results of studies using objective validation of water intake in adults. A Spanish beverage intake questionnaire for assessing water from beverages was tested for repeatability and validity by comparing consumption estimates with urine osmolality and 24-h volume. Using both the urine osmolality and the 24-h urine volume analysis, 66% of individuals were categorised in the same or adjacent quintile, demonstrating a reasonably good level of questionnaire validity. The questionnaire also demonstrated good repeatability; the difference between baseline measurement of total fluid intake and the measurement after 6 months and 1 year were not significant [32]. The Water Balance Questionnaire was adapted for use with Greek pregnant women and when compared to urine hydration indices, it was considered to be valid [33].

A recent and novel study has demonstrated that in an adult sample of men and women (aged 18–65 years) near a university in the US, a 7-day fluid record was a reliable method to estimate the distribution of daily water intake from fluids when compared to deuterium oxide disappearance [34]. This is the first time that a fluid intake questionnaire had been compared to total body water turnover.

It was noted in a review of the methodologies used to record fluid, beverage and water intakes at a population level in Europe, that none of the ten national surveys were validated for the assessment of water and fluid intakes [9]. Validation is essential for the establishment of dose–response relationships between water and fluid intakes and disease or other outcomes, and to develop robust intake recommendations [17].

Environmental factors such as temperature, altitude and humidity are known to affect fluid intake, so season should be a consideration when undertaking a population fluid intake survey [35]. There is some evidence that specific prompts for beverages and including water as a separate item in an assessment results in a more complete data collection [2].

Social desirability affects dietary recall across the population. Parents may want to reflect good parenting skills, children and adolescents may use food as a means of self-expression, and reports may be biased due to body-image beliefs [36]. It seems reasonable to assume that these factors are likely to impact the reporting of fluid intake.

An international panel on water quality recommended the use of a 4-day diary as the preferred method for collecting water consumption data, with a 24-h recall preferably repeated at least once, as the best alternative when this is not possible [35]. This recommendation followed a review of the literature on water intake and exposure studies.

There have been a number of recent studies comparing methods of obtaining total water intake indicating that results vary depending on the method used. An Indonesian study comparing fluid intake obtained by a 7-day fluid record versus a 24-h recall in adults (19–64 years) and adolescents (16–18 years) concluded that a 24-h food and fluid recall significantly underestimated total fluid intake when compared to a 7-day fluid record [37]. In Greek adults, water intakes obtained by a semi-quantified food and fluid questionnaire known as the Water Balance Questionnaire (WBQ) were higher than those obtained by a 7-day food and fluid record [38].

A Child Food and Beverage Questionnaire to assess intake of fruit, vegetables and sweetened foods and beverages was able to rank intakes rather than assess absolute intake in pre-school children estimated based on 3 × 24-h recalls [39]. Seven beverage survey questions had good correlation with data from 3 × 24-h recalls but the importance of asking times/day rather than servings/day in the questionnaires was noted [40].

In 11-year-old Danish children, the validity of self-reported fruit intake at school has been found to be superior to that of beverage intake. The use of opaque water bottles, which are re-filled throughout the day, makes quantification of consumption more challenging compared with easily counted pieces of fruit of a known size [41]. This demonstrates one of the challenges of assessing fluid intake; drinks such as water may be consumed in small mouthful amounts throughout the day, so an accurate recall of the amount consumed (rather than served) during the 24 h is required [9].

A brief 15-item beverage questionnaire to assess sugar-sweetened beverages and total beverage energy intake had good association with intakes obtained via three repeated 24-h recalls. In an adult population (> 18 years), the questionnaire underestimated total beverage intake by 9% on average [42]; it was shown to reflect changes in beverage consumption over time [43]. This type of questionnaire is particularly useful in health promotion or clinical situations to highlight risk and/or set behavioural goals.

In summary, beverages are often consumed throughout the day, rather than at discrete occasions, and this poses a challenge when assessing fluid intake. The assessment of fluid intake is a relatively new area of research. Studies to date have indicated that 24-h recalls significantly underestimate intake when compared to a 7-day diary [37]. A few beverage-specific questionnaires have been developed with promising early results [32, 33], and a recent study in adults has demonstrated the validity and reliability of a 7-day fluid record using total body water turnover [34].

## Portion size estimation

Portion size estimation is a recognised source of bias in dietary assessment. In a computer-based study, the effect of image size of sixteen food models and the presence of size cues (utensils and checked tablecloth) on accuracy of portion size estimation in children aged 8–13 years has been tested [44]. The portion size of food images were correctly classified in 60.3% of estimates when the foods were presented as (i) small graduated portion size images in one screen or (ii) by scrolling across large, graduated portion size images, one per sequential screen. The small graduated images took half the time to estimate the portion size and are thus recommended; the larger pictures led to overestimation of size [44]. In adults, it has been shown that eight pictures were better than four pictures for accurately estimating portion size [45].

A recent study in Cameroon was conducted to validate a book of food portion photographs in adults aged from 14 to 84 years and children aged from 3 to 13 years; children estimated 74% of the 556 foods to within 10% of the actual weight of the food; this was comparable to the 77% achieved by the adults [46]. This is in agreement with previous research in the UK which showed that children are able to assess portion size with similar accuracy to that of adults if provided with age-appropriate tools [47].

In a validation study of a web-based dietary assessment for self-reported fruit, fruit juice and vegetable intake, in 8–13-year-old Danish children, portion size estimation was the largest source of error [41]. In the validation of a 24-h self-completion questionnaire on beverage consumption in 7–9-year-olds, it was noted that the quantification of total beverage intake was flawed, so only group-level beverage consumption could be obtained [48].

In a child-adapted liquid and fluid diary in Belgian children aged 8–13 years, participants were provided with a marked cup to provide portion size information [49]. Such measures require further testing in children including whether the provision of a marked cup alters intake.

There is an argument that the portion size of fluids is more easily estimated than those of amorphous composite foods because the vessel they are drunk from can either be measured or estimated using a variety of household measures. Most purchased fluids will have the volume marked. The key issue is to capture what is actually drunk rather than what is served, e.g. if a child takes a 500-ml water bottle to school, it is important to consider how much of this was consumed. Clear instructions and a demonstration for study participants are essential to increase the accuracy of portion size estimation in children.

## Technology

Technology is changing the manner in which dietary intake data are collected by utilising alternative media. It is also advancing how details of consumption are captured, including portion sizes. Whilst these advances are both exciting and novel, they are mainly changing how dietary data are collected rather than what is collected, and rely on the traditional methods. Thus, compliance and motivation of the individual to accurately record their dietary intake remain of paramount importance.

Children and adolescents are particularly familiar with technology and their willingness to engage with technology supports the idea of using mobile applications for dietary assessment [50]. A novel study investigated the use of photographic food records obtained by a mobile application in two samples of children aged 3–10 years. The results indicated that with demonstration and practice these young children were able to use the mobile food record to capture their dietary intake [50]. In the first sample, one eating occasion was recorded; in the second, a 2-day record was obtained. This study offers potential for overcoming the issues with surrogate reporting in children [50]. A further study using 1 day of parental and other carers’ recording using a Tool for Energy Balance in Children (TECH) via a mobile phone in 3-year-olds was not able to accurately estimate energy intake or consumption of certain foods when compared to doubly labelled water and a FFQ, respectively [51]. A single day of recording is a major limitation of this study.

Another study has assessed the willingness of adolescents to take pictures of food and beverages consumed using a novel mobile food record [52]. This study showed that girls were more willing than boys to take images, and that breakfast was better captured than snacks or evening meals, thus demonstrating the need for training, reminders and entertainment when using this technology for dietary assessment [52, 53]. Other studies in adolescents have concurred that an app to record dietary intake is acceptable to adolescents [54]; prompts are needed to improve the quality of the data collected [55], the inclusion of barcode readers would be beneficial [56] and intrinsic motivation is required to obtain quality independent data [57]. As this is an area of active research and development, validation of many of these tools by either an objective criterion or by comparison to a reference method has not been undertaken.

A number of online dietary assessments have been developed, for example, the ASA24 in the US which is based on the USDA’s ‘Automated Multiple Pass Method’ [58] and a computerised recall was successfully used in adolescents in the HELENA (Healthy Lifestyle in Europe by Nutrition in Adolescence) Study [59]. When an early version of the ASA24 (with fewer probes for detail) was tested in children aged 8–13 years, the accuracy of the estimate of energy intake in younger children aged 8–9 years was lower than in older children aged 10–13 years [60]. An updated version of the ASA24, called ASA24-Kido, now has a mobile app and an option that permits multiple users (child, parent or carer) to record dietary intake across the day so it essentially moves with the child. A recent UK version of an automated 24-h recall has been developed; focus groups and usability testing indicated that it was suitable for all the age ranges tested (11–18 years and 19–64 years) [61]. This is consistent with findings of another online 24-h dietary recall tool when compared with an interviewer-led recall in 11–24-year-olds; similar mean energy and micronutrient levels were reported [62].

The authors are not aware of any studies investigating the use of technology to assess only fluid intake. Technologies that are able to accurately capture cup, beaker or vessel size will assist in portion size estimation; the use of an app to record intake on a smart phone may assist the problem of ongoing fluid intake throughout the day, e.g. by providing prompts at regular intervals throughout the day. These prompts could include whether anything has been drunk in the last time segment or has a water bottle been drunk and re-filled in this period.

## Conclusion

There is increasing interest in the importance of water and fluid intake to health, especially in children. More information is becoming available on fluid intakes globally, but there is a lack of consistency in the methods employed to assess intakes. Fluid assessment is difficult in any population and presents additional challenges in children and adolescents. Various methods exist, all of which have strengths and weaknesses; the ultimate choice of method depends on the research question and resources available. This issue has recently been addressed in a report that was published subsequent to the literature review undertaken [63].

If the research aim is to estimate total water intake in children accurately, obtaining concurrent accounts of both food and fluids consumed will give the most complete and accurate picture of total water intake, providing the method has good validity. For this to be achieved, there should be an emphasis on the importance of estimating fluid intake within dietary surveys.

If the research aim is to assess only fluid intake in children, a fluid-specific method, such as a diary or record, appears to be an optimal approach to provide an accurate estimate of intakes. Within each age group, certain considerations should be taken into account, for example, the recording of fluid intakes during school time. It is important to note that collecting data on fluid intake only means making an assumption about the water contribution of food to the diet. This is a potential source of bias, the impact of which will vary according to the reliability of this assumption in a given population.

Technology is rapidly advancing how intake data are collected and early research indicates that even young children are capable of using such techniques. Depending on the age of the child, the use of technology may overcome certain recording constraints, for example, the need for parent/carer involvement, or proxy reports. It also may reduce the burden of dietary assessment. To obtain reliable data, the motivation and commitment of the subject remain keys.
