# Correction to: Challenges in the assessment of total fluid intake in children and adolescents: a discussion paper

**DOI:** 10.1007/s00394-018-1761-7

**Published:** 2018-07-05

**Authors:** Janet Warren, Isabelle Guelinckx, Barbara Livingstone, Nancy Potischman, Michael Nelson, Emma Foster, Bridget Holmes

**Affiliations:** 1 FirstStop Nutrition Ltd, Oxford, UK; 2 Danone Nutricia Research, Route Départementale 128, 91767 Palaiseau, France; 3 Nutrition Innovation Centre for Food and Health, Ulster University, Coleraine, UK; 4 Office of Dietary Supplements, National Institutes of Health, Bethesda, USA; 5 Public Health Nutrition Research Ltd, London, UK; 6 Human Nutrition Research Centre, Newcastle University, Newcastle, UK

## Correction to: European Journal of Nutrition (2018) 57 (Suppl 3):S43–S51 10.1007/s00394-018-1745-7

In the original publication of the article, a mistake was introduced in affiliation of Dr. Michael Nelson; the correct affiliation is:

Michael Nelson

Public Health Nutrition Research Ltd, London, UK

